# Progressive Myoclonus Epilepsy: A Scoping Review of Diagnostic, Phenotypic and Therapeutic Advances

**DOI:** 10.3390/genes15020171

**Published:** 2024-01-27

**Authors:** Vincent Zimmern, Berge Minassian

**Affiliations:** Division of Child Neurology, University of Texas Southwestern, Dallas, TX 75390, USA; berge.minassian@utsouthwestern.edu

**Keywords:** progressive myoclonic epilepsy, Unverricht-Lundborg disease, Lafora disease, acid ceramidase deficiency, myoclonic epilepsy with ragged red fibers, neuronal ceroid lipofuscinoses, North Sea PME, therapy

## Abstract

The progressive myoclonus epilepsies (PME) are a diverse group of disorders that feature both myoclonus and seizures that worsen gradually over a variable timeframe. While each of the disorders is individually rare, they collectively make up a non-trivial portion of the complex epilepsy and myoclonus cases that are seen in tertiary care centers. The last decade has seen substantial progress in our understanding of the pathophysiology, diagnosis, prognosis, and, in select disorders, therapies of these diseases. In this scoping review, we examine English language publications from the past decade that address diagnostic, phenotypic, and therapeutic advances in all PMEs. We then highlight the major lessons that have been learned and point out avenues for future investigation that seem promising.

## 1. Introduction

Progressive myoclonus epilepsies, also described in the literature as progressive myoclonic epilepsies (PME), are a heterogeneous group of diseases characterized by variable-onset myoclonus, epilepsy, cerebellar involvement, and dementia. They progress towards varying levels of disability, and many, but not all, result in early death [1,2]. The scientific and clinical literature on the PMEs is quite large and investigators wanting to learn about the latest advances in this class of disorders are often left to search for monographs about individual disorders, which do not provide a view of how the field is advancing. This document is intended to fill this gap in knowledge by summarizing the past decade of clinical work on PMEs in the form of a scoping review. Scoping reviews, unlike systematic reviews, do not aim to resolve a specific clinical question and, as such, are not registered with PROSPERO and similar databases.

## 2. Scoping Review Methodology

**Figure 1:**
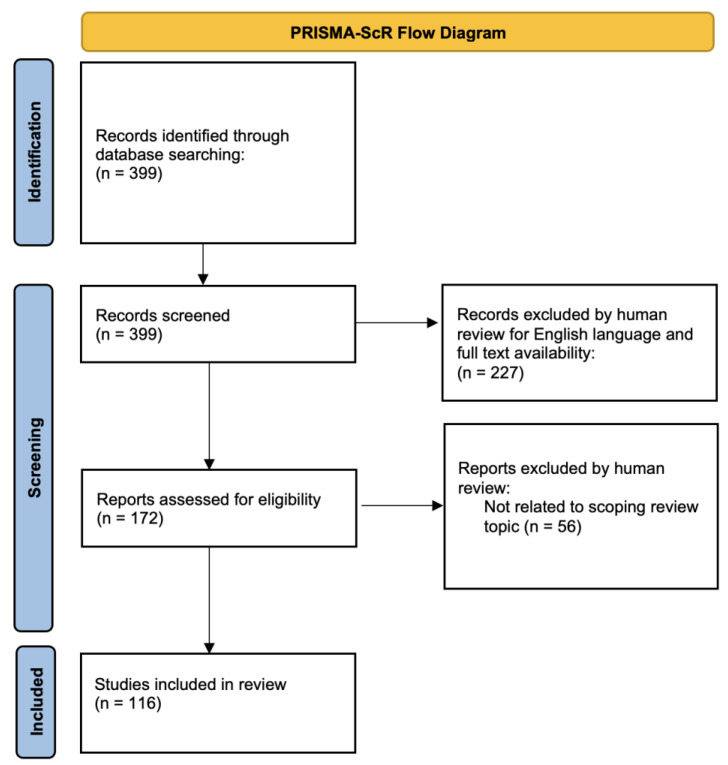
PRISMA-ScR flow diagram.

This scoping review aims to give readers a comprehensive overview of two aspects of the PMEs, namely (1) a diagnostic and phenotypic understanding of these disorders and (2) therapeutic modalities that are being pioneered in animal models or in human clinical trials. We include a discussion of pathophysiologic insights when addressing the PMEs as a whole but we do not discuss the pathophysiology of each of the individual PMEs as this would require a separate review. We cover the more common PMEs, listed in Table 1, in detail and present the publications for rare PMEs in tabular form for ease of access. This review limits itself to the past 10 years, specifically from 1 January 2013 to 11 January 2023. It also limits its range to publications in the English language that are available as full texts through PubMed. The search term “progressive myoclonus epilepsy” was used as a singular search term, which returned 399 articles. The exclusion of articles that were not in the English language and not available through PubMed led to the retention of 172 articles. After further manual review for relevance, a final 116 articles were retained for review. Please see Figure 1 for a PRISMA-ScR flow diagram.

This review is organized in two parts. The first part covers the generalized reviews and meta-analyses of these diseases as a whole and extracts some key messages about the progress made in our understanding of these disorders. The second part analyzes each of the principal diseases that make up the PMEs: Unverricht-Lundborg disease (ULD), Lafora disease (LD), neuronal ceroid lipofuscinoses (NCL), Sialidosis, North Sea PME, acid ceramidase deficiencies, myoclonic epilepsy with ragged-red fibers (MERRF), and rarer genetic PMEs.

## 3. PMEs: Novel Perspectives for This Class of Disease

### 3.1. Pathophysiologic Insights

The PMEs have very diverse genetic etiologies. This fact would suggest that there is no unifying pathophysiologic explanation for this class of diseases. Surprisingly, neuroinflammation appears to be a commonality in the pathophysiology of at least three of the more well-known PMEs, namely ULD (EPM1), LD (EPM2), and NCL [1]. Evidence for this comes from the various mouse models for these three PMEs. A murine model of ULD that lacks the *CSTB* gene (*Cstb−/−*) replicates the major features of the disease in humans [1]. Two-week-old *Cstb−/−* mice are asymptomatic but already show features of microglial activation, which then leads to neuroinflammation and neuronal loss [2]. In these same mice at 1 month of age, transcriptomic analyses of brain tissue reveal the upregulation of immune-related genes such as complement proteins, major histocompatibility complex class 1 (MHC-I), glial fibrillary acid protein (GFAP), cytokines, chemokines, and immunoglobulin receptors, among others [3]. This upregulation of immune genes in the brain is accompanied by the similar upregulation of chemokines and cytokines in serum, including the suggestion by the authors of this study that the chemokine Cxcl13 be considered as a biomarker for ULD [4]. In both the *Epm2a−/−* and *Epm2b−/−* knock-out mouse models of LD, there is a similar increase in genes that regulate the inflammatory response, the immune response, and phagocytosis [1]. Furthermore, these genes are further upregulated as the mice age and the disease symptoms worsen [5]. In certain mouse models of NCL (*Cln1−/−*, *Cln2−/−*, *Cln3−/−*), proteomic analyses of brain tissue reveal the upregulation of the inflammatory response and complement proteins [6]. A separate study of *Cln3−/−* mice shows that microglia activate reactive astrocytes to damage healthy neurons, suggesting that NCL-CLN3 may be a disease of astrocytic activation rather than a disease of the neuron itself [7,8]. The common finding of neuroinflammation, as a broad category of pathophysiology, suggests that anti-inflammatory medications could be an important therapeutic avenue, though the lack of human evidence of neuroinflammation in these disorders remains a gap in our knowledge [1,9].

Another possible commonality between the PMEs may be enhanced cortical excitability associated with giant somatosensory evoked potentials (SSEP) following N20 cortical potentials [10]. It is hypothesized that this electrophysiologic finding is the result of faulty post-excitatory inhibition, but the exact mechanism remains unclear. These giant SSEPs negatively correlate with blood oxygen levels (BOLD) on functional MRI (fMRI) compared to controls, but this study is limited by the small study population of three patients with ULD and one patient with KCNC1-related PME [10]. Further studies on this finding in PMEs are warranted and may provide insights into a common pathophysiology.

Other studies examining electrophysiological findings that are common to the PMEs have found abnormal blink reflexes [11]. The blink reflex (BR) involves an ipsilateral R1 and bilateral R2 and R2c responses. Compared to healthy subjects with normal latencies, patients with PMEs had abnormalities in the R2 and R2c components of their BR, including prolonged mean latencies and decreased amplitudes [11]. These findings correlated with the disease duration and use of multiple anti-epileptic medications. The authors suggest that these electrophysiologic abnormalities, seen more predominantly in patients with PME, correspond to the inhibition of the reticular formation of the brainstem [11].

### 3.2. Diagnostic and Phenotypic Advances

The past decade has seen significant progress in the diagnostic yield of genetic testing for PMEs. An Italian study of a cohort of 165 unrelated patients with PMEs revealed 47 with no genetic diagnosis [12]. Next-generation sequencing evaluations of 38 of these patients led to new genetic diagnoses in 16 patients, increasing the overall diagnostic yield of genetic sequencing in this cohort to 82% [12]. While several of the diagnoses involved known PME genes, other diagnoses involved genes not previously connected to PMEs and only known to cause developmental encephalopathies or progressive ataxias, further increasing the list of PME-related genes. Other studies have also demonstrated an increased diagnostic yield through next-generation sequencing, specifically thanks to whole-exome sequencing (WES). In a study of 84 patients with unsolved PME, WES led to the identification of novel disease-causing variants in 24 out of 78 (31%) unrelated individuals [13]. The yield was significantly higher in trio WES compared to singleton WES [13]. Among the newly identified disease-causing variants were variants in *NUS1*, *DHDDS*, and *ALG10*, all involved in dolichol-dependent protein glycosylation, disruptions of which had not previously been known to cause PMEs [13]. The study identified various other rare genes, along with genes not previously identified with PMEs, further confirming that most unsolved cases of PMEs are likely rare genetic diseases that will in due time be explained by ever more refined genetic sequencing methodologies [13,14].

### 3.3. Therapeutic Progress

Advances in therapies for PMEs have emerged through a better understanding of the best anti-seizure medications for seizures and myoclonus, along with improvements in gene therapy, enzyme replacement therapy, and other unique targeted approaches that will be discussed in later sections. An Italian case series of 11 patients with PMEs and a systematic review suggested a role for perampanel, an anti-epileptic medication, for the seizures and myoclonus of the various PMEs [15]. This medication was effective to reduce disability from myoclonus in 9 out of the 11 patients in their multicenter series and for half of the 104 patients evaluated in their systematic review [15]. Other commonly used anti-seizure medications (ASMs) include benzodiazepines, valproic acid, and levetiracetam [16,17].

Other treatment modalities that have been attempted for PMEs include dietary modifications such as the ketogenic diet and the modified Atkins diet; neuromodulation including repetitive transcranial magnetic stimulation, deep brain stimulation, and vagus nerve stimulation [18]; enzyme replacement therapies; protein therapies; mitochondrial tRNA modification; and gene therapies including viral, non-viral, and anti-sense oligonucleotides (ASO) [16,19,20]. Additional details about these modalities are discussed for each individual PME in subsequent sections of this review.

## 4. Unverricht-Lundborg Disease (ULD)

Unverricht-Lundborg disease (ULD) or progressive myoclonic epilepsy type 1 (EPM1) is an autosomal recessive disease that leads to action myoclonus, seizures, ataxia, and cognitive decline [21]. It is caused by biallelic mutations of the cystatin B gene (*CSTB*), with the most common mutation being the expansion of an unstable dodecamer repeat. Other rarer causes include compound heterozygous mutations, which seem to have gender-specific expressivity, with females showing rare seizures and sporadic myoclonus, while males exhibit a more severe phenotype with drug-refractory epilepsy and severe myoclonus [22].

### 4.1. Diagnostic and Phenotypic Advances

Longitudinal studies of ULD patients have revealed new insights in various domains related to the disease, including myoclonus, responses to transcranial magnetic stimulation (TMS), comorbidities and mortality rates, imaging findings, cognitive functioning, and autism.

Myoclonus is one of the most debilitating symptoms in ULD. In a small Polish study, myoclonus was noted in all patients and made it impossible for 10 out of 11 patients to move independently [23]. In light of the clinical significance of this symptom in ULD, more accurate and more automated assessments of myoclonus have been sought out. A Finnish study evaluated the human pose and body movement analyses of video recordings of 10 ULD patients and generated an automatic myoclonus rating scale (ARMS) [24]. This scale includes a jerk count during movement and a log dimensionless jerk score (LDLJ), keeping in mind that ‘jerk’ in physics refers to the third derivative of displacement with respect to time [24]. This ARMS correlated significantly with the unified myoclonus rating scale (UMRS), the clinical gold standard for myoclonus assessment, particularly for hand myoclonus but less for neck, trunk, and leg myoclonus [24]. Another Finnish contribution showed that surface electromyography and three-dimensional accelerometry, as measured in 23 ULD patients, correlated significantly with the UMRS scores and self-reported degrees of myoclonus [25]. This system accurately detected negative myoclonus in addition to the positive myoclonus of the disorder. These two studies suggest a future in which myoclonus monitoring at home and more accurate video-based analyses of myoclonus in the clinic may be the accepted standard of disease monitoring.

TMS-based studies of ULD have cast light on the pathophysiology and have improved disease phenotyping. For example, it has been hypothesized that neural processing progressively worsens in ULD as patients move from adolescence to adulthood. In a study of 8 adult patients, 6 adolescent patients, and 10 adult controls, patients with ULD showed a mild or missing repetition suppression (RS) phenomenon in response to the TMS of the motor cortex, in comparison with controls who showed healthy RS [26]. Abnormal RS correlated with the myoclonus severity and worsened with patient age. The authors suggest that impaired thalamocortical relays or impaired inhibitory tone leads to these abnormalities in RS, which could be used as a biomarker in future trials [26].

Previous work showed that TMS-induced long-interval intracortical inhibition (LICI) was normal in patients with ULD, but that short-interval intracortical inhibition (SICI) was reduced [27]. A study of 19 patients with ULD (15 with biallelic expansion repeats, 4 compound heterozygotes) and 7 healthy, age- and sex-matched controls showed that patients had significantly less SICI compared to controls [27]. However, neither LICI nor SICI was associated with the clinical severity of the disease. The study authors suggest that LICI and SICI may be useful in future trials as markers of deranged GABA-ergic inhibition, which is known to contribute to the pathophysiology of ULD.

As far as the comorbidities and mortality rates of ULD are concerned, we know from a single-center longitudinal study in Finland that patients with ULD tend to suffer from previously unappreciated comorbidities, including external injuries (e.g., ankle fractures), diabetes, and depression [28]. This study complements another Finnish study of 135 patients with ULD that demonstrated comparable survival rates with age-matched controls up to the age of 40, after which patients with ULD had worsening survival rates [29]. It should be noted, however, that approximately 10% of the cohort had mild disease progression, with some patients retaining functional independence well into their 50s [29].

Other than non-specific MRI atrophy findings, distinct imaging findings in ULD have been difficult to identify. In a Finnish study of 18 patients who had undergone clinical and neuropsychological evaluations, magnetic resonance spectroscopy (MRS) and magnetic resonance imaging (MRI) found significant cerebral metabolic changes in lactate, N-acetyl-aspartate (NAA), and choline that were noted in the basal ganglia, thalamic nuclei, insula, and occipital areas [30]. Changes in the right insula, basal ganglia, and thalamus were associated with the impairment of psychomotor and executive function [30]. In a study of a single patient with genetically confirmed ULD and alcohol-responsive myoclonus, brain MRI showed mild cerebellar atrophy, while fluorodeoxyglucose position emission tomography (FDG-PET) revealed hypometabolism prominently in the posterior brainstem, thalami, and frontal and parietal lobes [31]. The study authors suggest that this hypometabolism may be shared across PMEs, including Lafora disease, but that this finding will require further study [31].

Several studies have focused on better understanding the impact of ULD on cognition. A Finnish study of 68 patients with ULD demonstrated impaired cognitive performance compared to healthy controls [32]. ULD patients in this study had below-average scores on the verbal intelligence quotient and performance intelligence quotient subscores of the neuropsychological assessment. Their executive function and psychomotor speed were also impaired compared to controls, although the authors acknowledged that the timed tests may have been impacted by the severity of the myoclonus. As regards the possibility of an association between ULD and autism, there is a single case report of several patients at a single center who presented with features of autism [33]. However, it should be noted that these children did not undergo genetic testing to confirm the ULD, so this association remains unclear.

### 4.2. Therapeutic Progress

Several studies have evaluated seizure management in ULD patients. A study looking at the long-term use of anti-seizure medications (ASM) in a cohort of 20 Finnish patients with ULD found that all were on polytherapy, with freedom from generalized tonic-clonic (GTC) seizures in 14 of these patients [34]. All patients were on valproic acid. While additional ASMs were often associated with significant adverse effects, the addition of piracetam, topiramate, or levetiracetam led to a subsequent improvement in myoclonus [34]. In the previously mentioned Polish study of 11 patients with ULD, the authors noted a similar improvement in GTC seizures with valproic acid and an improvement in myoclonus with add-on clonazepam, levetiracetam, or topiramate [23]. Consistent with our earlier discussion of perampanel (PER) as a useful adjunctive medication for PMEs generally, evidence is accumulating in favor of PER for both seizures and myoclonus. A French study of adjunctive PER in 12 patients with ULD noted a robust decrease in myoclonus in 10 patients (80%) and seizure cessation in 6 patients (50%). A weight gain of 6–8 kg and psychological and behavioral side effects were both noted in 50% of patients, which led the study authors to recommend the close observation of patients while on this medication [35]. A single case report from Japan also reports a significant reduction in myoclonus after the initiation of adjunctive PER, with an associated robust decrease in the amplitude of a giant SSEP that was evaluated serially over the course of a decade [36].

Brivaracetam (BRV) has been studied in ULD for both myoclonus and seizures. Two prospective, multi-center, double-blind phase 3 trials of BRV in ULD patients demonstrated no effect on action myoclonus [37]. However, the action myoclonus scores were highly variable, suggesting that the myoclonus scale may not have been the optimal tool to evaluate the severity of myoclonus. In an open-label extension trial of BRV, 92.6% of ULD patients developed treatment-emergent adverse effects (TEAE), while 17% of ULD patients discontinued the medication due to drug-related TEAE [38]. Despite these statistics, the authors conclude that BRV is well tolerated by patients with ULD.

## 5. Lafora Disease (LD)

Lafora disease (LD) is a fatal form of PME characterized by progressive myoclonus, refractory seizures, and cognitive decline. It is caused by biallelic variants in essential glycogen metabolic enzymes laforin, encoded by *EPM2A*, and malin, encoded by *NHLRC1* (also called *EPM2B*) [39]. These mutations lead to the generation of abnormal glycogen branches in a subgroup of glycogen molecules, leading them to precipitate and accumulate into polyglucosan bodies called Lafora bodies (LBs), which in turn appear to drive a neuroinflammatory response [40].

### 5.1. Diagnostic and Phenotypic Advances

Several genotype–phenotype studies and natural history studies of LD have been published over the past decade and cast some light on how the underlying genetics can predict the natural evolution of the disease. An epidemiologic study identified a prevalence rate in Germany of 1.69 per 10 million people and highlighted the importance of whole exome and gene panel testing in undiagnosed cases of PMEs, as eight cases revealed novel variants [41]. A recent systematic review of known pathogenic variants in both *EPM2A* and *NHLRC1* identified 250 cases over 70 articles [39]. It found a distribution of 56% of cases with *NHLRC1* mutations and the remainder of the mutations being in *EPM2A*. After multivariable Cox regression analysis, the genotype corresponding to biallelic protein-truncating (PT) variants in *NHLRC1* was associated with shorter survival and a trend towards a higher probability of loss of autonomy, while the genotype corresponding to biallelic p.Asp146Asn variants in *NHLRC1* had a more favorable prognosis. These results clarify the outcomes of an earlier Italian long-term follow-up study of patients with LD, which followed 26 patients over an average follow-up period of 11.48 years [42]. The study identified an overall worse disease prognosis in patients with *EPM2A* mutations, rather than *NHLRC1* mutations, but found that the biallelic *NHLRC1* p.Asp146Asn variant genotype was associated with the delayed onset of disabling symptoms, as opposed to a more favorable prognosis. This is consistent with the findings of a separate study on a milder form of Lafora disease, which identified six patients from a cohort of 23 with a milder phenotype [43]. The characteristics of this milder phenotype include a delayed age of onset of disease, a lower neurologic disability score at 4 years after onset, less severe seizures, and a lower likelihood of showing both of the characteristic electrophysiologic findings in LD (i.e., photo-paroxysmal response on electroencephalography and giant SSEP). All six of these patients carried *HNLRC1* mutations and five of the six carried either homozygous or compound heterozygous p.Asp146Asn mutations [43].

A natural history study of LD published by Italian researchers evaluated 298 genetically confirmed cases and noted an average age at disease onset of 13.4 years, with survival noted in 272 cases to be 93% at 5 years of age, 62% at 10 years, and 57% at 15 years [44]. The median time to loss of autonomy was 6 years, and the median survival time was 11 years. Univariate and multivariate Cox regression models with mixed effects identified that “Asian origin” and “age at onset < 18 years” were negative prognostic factors, while there were no associations between genotype and survival or disability. This latter finding appears to contradict the systematic review results mentioned previously [39].

A few case reports have added insights into the phenotypic range of LD. A Turkish patient with genetically confirmed LD developed signs of parkinsonism (i.e., rigidity, bradykinesia) by age 11 after having first developed GTC seizures [45]. Only one prior patient with LD, aged 53 at the time, has ever been reported to have developed parkinsonism [45]. Whole exome sequencing continues to grant insights into the pathophysiology of LD as novel mutations reveal insights into the functions of malin and laforin [46,47].

Several studies have looked for imaging and electroretinogram (ERG) abnormalities that could either serve as biomarkers or, at the very least, shorten the duration of the diagnostic process for patients and their families. A case report from China identified a patient with LD who was found to have non-specific global brain atrophy on MRI at the time of diagnosis [48]. This finding differs from a 2006 cohort imaging study of patients with LD, which identified minimal MRI brain volume changes but significant MRS changes in frontal cortex and basal ganglia [49]. For now, there does not appear to be a robust and consistent brain MRI finding for LD. Attempts have also been made to identify biomarkers through studies of the retina. A study of four patients with LD identified a normal retinal structure, with good visual acuity, normal retinal examination, and normal optical coherence tomography. However, ERG did reveal abnormal retinal function in all patients, evidenced by a reduced b/a ratio of scotopic ERG in three out of the four patients, along with generalized cone system dysfunction with inner retinal (i.e., bipolar cell) involvement in all four patients [50]. This was consistent with a prior finding of bipolar cell atrophy from Lafora body accumulation seen on histology. This finding was further confirmed in a subsequent study of six patients with LD—mild to severe generalized cone dysfunction of the retina was confirmed on ERG in all patients [51]. Interestingly, cone and rod dysfunction correlated with both the disease duration and the type of mutation. Given these early results, one can imagine that ERG may become an important element of the workup for LD, especially in diagnostically challenging cases.

### 5.2. Therapeutic Progress

LD remains incurable but significant effort is being made to provide symptom relief while working on definitive cures. Treatment efforts for LD can be categorized into (1) the optimization of anti-seizure and anti-myoclonus medications, (2) the repurposing of existing medications, (3) antibody–enzyme fusion (AEF) to degrade LBs, (4) strategies to reduce glycogen synthase, and (5) new vectors for gene therapy, including cationic lipoplexes, all of which have been developed either in vitro or in mouse models, without associated clinical trials for the time being. Combinations of these strategies that incorporate genetic repair with medications that are known to alleviate symptoms may prove useful in the near future [52].

We have previously discussed perampanel’s use in PMEs as a group of disorders. In a small study of 10 patients with genetically confirmed LD, perampanel was added to the patient’s pre-existing medications, with follow-up at 3 and 9 months [53]. While three patients withdrew due to inefficacy or side effects, four had significant seizure reductions (>74% compared to baseline) and another seven had significant improvements in myoclonus. No significant improvement was noted in cognition or overall disability. Overall, it appears to be an important addition to the armamentarium of the neurologist when treating both the seizures and myoclonus of this condition.

Several medications have been repurposed for use in LD, including metformin, 4-phenylbutyric acid (4-PBA), trehalose, sodium selenate, propranolol, and epigallo-catechin gallate (EGCG). Several of these medications—metformin and trehalose specifically—are autophagy flux modulators and are thought to be helpful in LD because impaired autophagy is a part of the pathophysiology of the disease [54]. Metformin, the most widely used medication for type 2 diabetes, activates adenosine monophosphate kinase (AMPK), which in turn inactivates glycogen synthase. Since glycogen synthase is implicated in the generation of LBs, treatment with metformin would be suspected to at least stop the progression of LB formation. A 2016 study of metformin in a *Epm2b−/−* mouse model showed a decrease in the accumulation of LBs as well as polyubiquitin protein aggregates in the brain compared to control mice [55]. The study also noted fewer features of neurodegeneration (i.e., neuronal loss, reactive gliosis) and better results in the neuropsychological testing of the mice. Follow-up studies of metformin looking at seizure reduction did find a decreased seizure frequency and duration as well as improved mortality in both *Epm2a−/−* and *Epm2b−/−* mice whose seizures were provoked by pentylenetetrazol (PTZ) [56,57,58]. These findings led to the designation of metformin as an orphan drug for the treatment of LD by the European Medicines Agency (EMA) in 2016 and the United States Food and Drug Administration (FDA) in 2017. A clinical study of 10 patients with LD was inconclusive, with three patients showing some temporary benefit, but this was likely because many of the patients had advanced disease [59].

The molecular chaperone 4-PBA has also been intensely studied for use in LD and seems to have similar effects to metformin. These include similar findings of fewer LB and polyubiquitin protein aggregates, decreased neurodegeneration, improved neuropsychological testing results, a reduced seizure frequency and duration, and improved mortality in *Epm2b−/−* mice [55,56,58]. Another molecular chaperone and autophagy flux modulator, trehalose, has been studied in various animal models of LD. Early supplementation of trehalose in *Epm2a−/−* mice improved their susceptibility to PTZ-induced seizures but did not have an impact on LB formation [60]. A similar study on zebrafish (which reproduce the LD phenotype) showed that the early administration of trehalose reduced neuronal excitability, decreased the seizure frequency, and improved motor function [52]. This suggests that the induction of autophagy by trehalose may be an important direction for future LD therapies.

Given that LD has a neuroinflammatory component, studies have looked into the potential of anti-inflammatory treatments. Studies have previously shown that heat shock proteins (HSPs), which act as molecular chaperones, minimize stress-induced damage. These HSPs, which coordinate the heat shock response (HSR), are controlled by a master transcriptional regulator, heat shock factor 1 (HSF1). Laforin and malin function upstream of HSF1 and are important for the activation of the HSR. *Epm2a−/−* and *Epm2b−/−* mice have reduced levels of HSF1, suggesting a compromised HSR [61]. Treatment with dexamethasone, a synthetic glucocorticoid, partially restores the levels of HSF1 and its downstream target molecules. Dexamethasone was able to improve neuroinflammation and susceptibility to seizure in these mice but did not lead to any improvement in LB formation [61]. Other anti-inflammatories—propranolol (a β-adrenergic antagonist) and EGCG (a green tea extract)—have been shown to improve neuronal disorganization, astrogliosis, and microgliosis in the hippocampi of *Epm2b−/−* mice after 2 months of treatment [62]. Selenium, a strong antioxidant, also seems to improve both motor and memory deficits in *Epm2b−/−* mice [63]. After treatment with sodium selenate, the PTZ-induced seizure sensitivity improved, as did neuronal degeneration and gliosis.

Because the formation of LBs seems to be at the core of LD pathophysiology, researchers have looked for ways to clear these polyglucosan bodies. One approach that seems very promising is called antibody–enzyme fusion (AEF). The strategy involves an antibody–enzyme complex, composed of a cell-penetrating antibody fragment fused to human pancreatic amylase. This fusion, called VAL-0417, degrades LBs in vitro and significantly reduces the LB burden in vivo in *Epm2a−/−* mice [64]. Metabolomic analysis shows that, after treatment with VAL-0417, *Epm2a−/−* mice have an identical metabolic profile to wildtype mice, suggesting a dramatic reversal of the disease process [64].

Disrupting glycogen synthesis by suppressing glycogen synthase (GS) from the early embryonic stages in a mouse model of LD prevented the formation of LBs and thus the manifestations of LD [65]. Further work with a murine model of LD with inducible suppression of GS showed that GS suppression in adult mice blocked the formation of new LBs and reduced the overall glycogen aggregate burden [66]. In the brains of these mice, early but not late GS suppression stopped the accumulation of LBs. This work confirms that the suppression of GS is a viable approach to treating LD but depends on a critical therapeutic window and early intervention. Further work in this direction, aiming at downregulating glycogen synthase in the brain, led to the development of an antisense oligonucleotide (Gys1-ASO) targeting the mRNA of brain-expressed glycogen synthase 1 gene (Gys1) [40]. After the intracerebroventricular injection of this ASO, mice were analyzed for pathologic signs of LD. The ASO prevented LB formation in mice that had not yet formed them, while, in older mice with pre-existing LBs, the ASO stopped the further progression of the LB burden. The disruption of LB formation then led to trends towards the correction of the neuroinflammatory cascade in these mice. This work on glycogen synthase inhibition, taken as a whole, is a very exciting path forward for the treatment of LD, if implemented at a sufficiently early stage of the disease.

Another exciting development has been the production of cationic liposomes to serve as a nonviral gene delivery vector for LD gene therapy. The study authors generated two types of liposomes for the delivery of the laforin gene to two cell types (HEK293 and neuroblastoma cells) [67]. The nanosized liposomes showed excellent transfection efficiency and minimal cytotoxicity. Importantly, Western blot tests after transfection confirmed the cellular expression of laforin, while a glucan phosphatase assay confirmed the biological activity of the expressed laforin. This paves the way for the use of cationic liposomes for gene therapy.

## 6. Neuronal Ceroid Lipofuscinoses (NCL)

The neuronal ceroid lipofuscinoses (also called Batten disease) are a complex, genetically diverse group of disorders with a broad phenotypic spectrum [68]. What they have in common is neurodegeneration and the intracellular accumulation of an auto-fluorescent lipopigment (ceroid lipofuscin) [69]. The NCLs have undergone several classification schemes, with a recent expert opinion recommending nine sub-types (CLN1-CLN8 disease, CLN10 disease). With advances in genomic sequencing, it has become clear that neither the causative variants nor the age of onset (which continues to be used for the broad description of these disorders, e.g., juvenile, infantile, late-infantile, adult-onset) can always predict the genotype [69]. While some continue to recommend rational diagnostic testing based on the age of onset and phenotype, others recommend broader genetic testing, including whole exome sequencing [70].

### 6.1. Diagnostic and Phenotypic Advances

The NCLs are generally considered to be members of the PMEs because they tend to feature progressively worsening myoclonus and seizures. However, a recent paper has cast doubt on this widely held view, by showing that myoclonus is not as common in juvenile NCLs (JNCL) as was once believed [71]. This study, using data from a natural history study of JNCL, included 86 children and found that 86% (74 patients) experienced at least one seizure, mostly GTC in semiology. Myoclonic seizures were infrequent, being reported in only 16% of the study population. The study authors conclude that the myoclonic feature in JNCL is not as significant in this population as has previously been believed [71].

Most publications on the NCLs that focus on the PME aspect of the disease have focused on establishing the genotype–phenotype correlation. CLN6 disease manifests with ataxia, seizures, visual loss, and developmental regression. A single-center cohort study of 97 patients with CLN6 disease identified 86 late-infantile, 8 juvenile, and 3 adult-onset cases [68]. The age at referral ranged broadly from six months to 33 years of age, and 45 distinct genotypes were identified, 24 of which were novel. These facts underscore the broad range of ages at onset and the equally broad mutational spectrum of this disease. Another study of 11 patients with CLN6 disease identified a consistent pattern of learning delays in children that preceded the onset of seizures, visual loss, and myoclonus [69]. However, in three patients with teenage or adult onset of disease, the classic PME phenotype emerged prominently and early, with a learning disability preceding the onset of PME in one patient. Consistent with our previous discussion of giant SSEPs and the presence of a photo-paroxysmal response (PPR) in PMEs as an indicator of cortical hyperexcitability, the SSEPs were very enlarged and a PPR was present in all these patients [72].

### 6.2. Therapeutic Progress

While our scoping review search did not return any articles specifically addressing the topic of therapies for NCLs, there is a growing body of literature on viral-mediated gene therapies and enzyme replacement therapies for the various disorders within this group. Enzyme replacement for CLN2 Batten disease (cerliponase alfa) was approved in 2017, setting the stage for similar therapies for the remainder of the NCLs [73]. For more information on the therapies in the pipeline for the NCLs, we recommend Johnson et al.’s review [73].

## 7. Sialidosis

Sialidosis is a rare cause of PME, caused by a deficiency of α-N-acetyl neuraminidase caused by mutations in the neuraminidase 1 gene (*NEU1*) [74]. Clinically, it presents in two forms. Sialidosis type 1, sometimes called “cherry-red spot myoclonus syndrome”, is relatively mild and usually occurs in the second decade of life with the two symptoms mentioned in its other moniker. Sialidosis type 2 has both infantile and juvenile-onset forms and presents similarly to type 1 but, in addition to myoclonus and macular cherry red spots, also features coarse facial features, corneal clouding, and dysostosis multiplex [74].

### 7.1. Diagnostic and Phenotypic Advances

Studies of this rare condition are limited. A small study from China identified four patients that were evaluated retrospectively with genetically confirmed sialidosis type 1 [75]. Three of the four presented with PME. All these patients had abnormalities in optical coherence tomography (OCT), fundus autofluorescence (FAF), and visual evoked potentials (VEP). These findings have limited generalizability given the small study but suggest that optical tests (OCT, FAF, VEP) may help to shorten the diagnostic process and fit logically with what is known about the disease, namely that metabolic breakdown products accumulate in the nerve fiber and ganglion cell layers of the retina [75]. These ocular findings also align with a previous case report with PME from sialidosis type 1 [76].

Follow-up studies of patients with type 1 sialidosis are few but very revealing. A case series of four unrelated patients with type 1 sialidosis showed that these patients maintained normal cognition while their motor and speech capacities worsened, over a maximal follow-up time of 30 years. Perampanel was found to be useful for both myoclonus and GTC seizures, while MR imaging of these patients revealed new findings of bilateral gliosis of the cerebellar folio and the occipital white matter [77]. A case report of a Japanese patient with sialidosis type 1 who was followed over 41 months noted non-specific diffuse brain atrophy [78].

### 7.2. Therapeutic Progress

Consistent with our theme of perampanel use in PME, a case report of perampanel in a patient with genetically confirmed sialidosis who was experiencing near-continuous myoclonus describes the remission of myoclonic seizures within one month of starting the medication as an adjunctive gradually titrated to 10 mg/day [74]. The study authors also noted some improvement in his neurological and cognitive function over the next 20 months of being on the medication. Similar improvements were noted in a patient with type 1 sialidosis from Hong Kong, whose dose was gradually titrated to 6 mg/day [79].

## 8. North Sea PME

North Sea PME is a rare autosomal recessive disorder that originates from mutations in the Golgi SNAP receptor complex 2 (*GOSR2*) gene. This gene encodes membrin, a SNARE protein on the cis-Golgi membrane. Mutations in this essential protein for the fusion of COPII vesicles from the endoplasmic reticulum (ER) onto the cis-Golgi membrane ought to have significant systemic effects on multiple organs, but the effects of these mutations seem to be limited to the central nervous system (CNS) [80]. In this regard, North Sea PME is similar to other PMEs like LD and NCLs in that the autophagy lysosomal pathway (ALP) is implicated in the pathophysiology of the disease. Patients with this disorder tend to originate from countries bordering the North Sea, reflecting the name [81].

### 8.1. Diagnostic and Phenotypic Advances

This rare PME seems to have a very consistent clinical and electrophysiologic presentation. This view is bolstered by a study of 12 patients with genetically confirmed North Sea PME (i.e., *GOSR2* mutations) [81]. The clinical presentation was remarkably consistent across the study cohort: early-onset (i.e., 2 years of age) ataxia, followed by myoclonic seizures at the age of 6.5, scoliosis by adolescence, and premature death by the third or fourth decade of life. Other skeletal abnormalities in these patients included pes cavus and syndactyly. All patients had elevated creatine kinase levels but had normal muscle biopsies. The disease course was a progressive decline, leading uniformly to wheelchair dependence. The study authors noted a likely founder effect as all 12 patients, each originating from countries neighboring the North Sea, carried the same homozygous mutations (c.430G>T, G144W). The study authors also noted the significance of scoliosis as an important diagnostic clue in this PME.

Despite this study revealing the relatively uniform presentation of North Sea PME, there are additional studies pointing to a wider genotype–phenotype spectrum than previously thought [82]. At least two studies have demonstrated a phenotype of muscular dystrophy associated with *GOSR2* variants [83,84]. Moreover, developmental delay and dystonia have also been reported as part of the phenotypic spectrum of North Sea PME. Specifically, a patient who was compound heterozygous for the *GOSR2* splice site variant c.336+1G>A and a novel c.364G>A, p.Glu122Lys missense variant presented with a global developmental delay and epilepsy at the age of 2, and clonazepam-responsive myoclonus at age 8—which is a radical departure from the typical sequence of symptom onset for North Sea PME. Another patient, homozygous for the founder mutation in *GOSR2* (c.430G>T, G144W), only manifested mild fine motor development and infection-triggered seizures that remitted after the introduction of levetiracetam. She then went on to develop the movement disorder associated with North Sea PME, but did so at an atypically slow rate, culminating in ataxia and dystonia. These patients not only expand the mutational and phenotypic spectrum of North Sea PME but also suggest that *GOSR2* should be added to the list of monogenic causes of dystonia, epilepsy, and global delay [83,84]. This conclusion is further buttressed by a study that identified a novel variant that expanded the *GOSR2* mutational spectrum [85]. The new variant was identified in a 61-year-old Caucasian woman who had developed ataxia at age 2, with transient episodes of worsening motor function with febrile illness or infection-related triggers. She then developed generalized action myoclonus and epilepsy at age 14. Over the years, she developed scoliosis and areflexia. She was heterozygous for the *GOSR2* c.430G>T variant—further sequencing of *GOSR2* exons revealed a novel in-frame 3-base-pair deletion causing the deletion of lysine (p.K164del) in an importantly conserved portion of the protein structure. Her brother was found to be a heterozygous carrier of the classic variant and had typical cervical dystonia, though it remains unclear whether the isolated dystonia in this family member was due to *GOSR2* heterozygosity, as the patient may have had other dystonia-related gene variants that were not investigated in modern dystonia gene panels. This new variant expands the *GOSR2* mutational spectrum and suggests that patients with North Sea PME may have slower progression and a longer lifespan than was previously believed. Moreover, this study introduces the possibility that patients with isolated dystonia may be heterozygous for *GOSR2* variants.

### 8.2. Therapeutic Progress

There are no cures for North Sea PME and the management of this disorder has been symptomatic care. North Sea PME is typically refractory to anti-seizure medications. A ketogenic diet, or a less stringent version of it, namely the modified Atkins diet, has been shown to be helpful for many seizure syndromes. A prospective, open-label study of the modified Atkins diet in North Sea PME evaluated four patients and assessed patients at baseline and 3 months after diet initiation [86]. The primary outcome was a quality-of-life metric, with secondary outcomes being seizure frequency and blinded rated myoclonus severity. The diet was well tolerated, and all patients completed the 3 months of dieting. Health-related quality of life improved considerably for one patient and remained sustained during long-term follow-up. This primary outcome remained unchanged in the remaining three patients and those patients did not continue with the diet. Overall, given the small numbers in this study, one cannot be confident that the modified Atkins diet will help, but it could be attempted in this patient population as a small percentage seem to be respond favorably.

Deep brain stimulation (DBS) is a neurosurgical procedure that implants a neurostimulator in certain brain areas—the targeted stimulation of these areas leads to symptom improvements in Parkinson’s disease, essential tremors, and other movement disorders. DBS was attempted for North Sea PME in three patients from the same white Afrikaans family from South Africa with the classic homozygous *GOSR2* mutation [87]. The DBS was placed in the caudal zona incerta in all three patients before they came to the attention of the study authors. The patients experienced a reduction in GTC seizures and two of the three experienced a reduction in involuntary movements. One patient had an improvement in gait and stance when the stimulation was applied. Overall, this suggests that DBS may play a role in the management of this PME, but more research is needed.

## 9. Acid Ceramidase Deficiencies

Acid ceramidase (AC), encoded by the *ASAH1* gene, is an enzyme implicated in the metabolism of ceramide. Mutations in *ASAH1* cause two different disorders: Farber disease (FD) and spinal muscular atrophy combined with progressive myoclonic epilepsy (SMA-PME) [88]. It is thought that acid ceramidase is important for lysosomal positioning in the cell and subsequent neuronal development, but the exact pathophysiology of these disorders remains unclear [88]. Farber disease is a lipid and lysosomal storage disorder, also known as Farber’s lipogranulomatosis, because the disorder is characterized by diffuse subcutaneous nodules from lipid accumulation. In the classic variant of FD, also called type 1 FD, the lipogranulomas are accompanied by joint contractures and voice hoarseness, making up a typical symptomatic triad. In total, there are seven subtypes of FD, and type 5 is the “neurological progressive” type, which manifests with seizures and neurological deterioration. FD, despite being protean in its manifestation, is not classically considered a PME and therefore the focus will be on the other form of acid ceramidase deficiency, SMA-PME. SMA-PME was originally classified as a subtype of SMA but, over time, as more cases were described, the similarity with FD became more obvious [89]. The genotype–phenotype correlation is poor as there are documented cases of patients with FD and SMA-PME with the same mutation [89]. There are no treatments for this PME, and the clinical focus is on symptom management and supportive care.

### 9.1. Diagnostic and Phenotypic Advances

The genotype–phenotype spectrum of SMA-PME is an expanding area of clinical research. The most common causative variant is a biallelic mutation, p.T42M in *ASAH1* [90]. This variant is associated with the presentation of lower motor neuron disease (i.e., weakness, hyporeflexia) preceding the onset of myoclonus and seizures. A rare phenotype associated with this variant, reported in a 6-year-old Turkish girl, is eyelid myoclonic status epilepticus [91]. In a study of three Iranian patients, exome sequencing in combination with testing to evaluate survival motor neuron 1 (*SMN1*) and 2 (*SMN2*) copy numbers to rule out SMA revealed new causative variants in *ASAH1* [90]. In a study of six new patients with the hallmark features of SMA-PME—namely lower motor neuron disease, tremor, and ataxia without the triad of FD—five of the six patients carried at least one of the known SMA-PME variants in *ASAH1* [92]. In the same study, a review of 30 cases showed that patients homozygous for the common c.125C>T variant presented in the first decade of life with limb-girdle weakness, while patients with the c.456A>C variant experienced sensorineural hearing loss. Another study from Italy of three patients with various pathogenic mutations in *ASAH1* confirmed many of these findings but expanded the phenotypic spectrum by including imaging and electrophysiologic findings [93]. EEG was consistent with a PME, including generalized spike-and-wave discharges, evidence of positive and negative myoclonus, and significant photosensitivity. Surprisingly, SSEP were unremarkable in all patients. Abnormal VEP and auditory evoked potentials (AEP) suggested that the patients had disrupted visual and auditory brain pathways, though structural abnormalities on brain MRI were only noted in one of the three patients. Clearly, the small number of patients in this study is insufficient to generalize to this disease class, but it is an important start in understanding the natural history and characteristics of this rare disease.

### 9.2. Therapeutic Progress

Care for AC deficiencies remains symptomatic. Emerging therapies for FD and SMA-PME include gene therapy and enzyme replacement therapy (ERT). Early studies on gene therapy for FD showed that onco-retroviral-mediated gene transfer in patient-extracted cells led to the expression of acid ceramidase (ACDase) with the resulting normalization of ceramide levels [89]. Non-transfected cells also normalized their ceramide levels because of the local effect of ACDase secreted into the local tissue, which makes this even more promising as a strategy. This same approach was successful when using lentiviruses with hematopoietic stem cells. This approach, when applied to the FD P361R mouse model, led to an increased lifespan from 9 weeks to 16.5 weeks [89]. However, there are no current clinical trials for gene therapy in FD and SMA-PME. A gene therapy approach has proven successful in a FD P361R mouse model, with prolonged survival and clearance of inflammation in peripheral tissue [94]. This gene therapy was attempted in 2023 in a compassionate care situation for a patient with advanced SMA-PME—the patient died 6 days after gene therapy. This therapeutic attempt and details on the patient’s demise are to be published at a future date [95]. Successful gene therapy has been developed and obtained FDA approval for SMA type 1 (i.e., onasemnogene abeparvovec), which may build a case for use in SMA-PME, but there are no existing case reports of its use for these patients [96]. ERT with recombinant human acid ceramidase (rhACDase) is being developed [89,96,97]. Given the success of ERT for CLN2 Batten disease, there is hope that similar therapies for the AC deficiencies will also be successful [97].

## 10. Myoclonic Epilepsy with Ragged Red Fibers (MERRF)

MERRF syndrome is one of the most common mitochondrial disorders due to point mutations in mitochondrial DNA (mtDNA) [98]. The main point mutation is the m.8344A>G mutation in the tRNALys gene of mtDNA (also called the MT-TK gene). This mutation leads to the disruption of the translation of proteins encoded by mtDNA, which then leads to the disruption of the electron transport chain (ETC) complexes of the mitochondria. In turn, this results in impaired respiratory function and dysfunction of cellular energy production [98].

### 10.1. Diagnostic and Phenotypic Advances

The phenotypic spectrum of MERRF, like many of the mitochondrial encephalomyopathies, is quite varied, as many different tissue types may be involved. In addition to the presentation of PME and weakness, there are reports of symmetrical lipomatosis, including cervical lipomatosis [99], spinocerebellar-like ataxia, severe Leigh syndrome, sensorineural deafness, myopathy, optic atrophy, and chronic progressive external ophthalmoplegia (CPEO) [100]. The mutational spectrum of MERRF continues to expand [101]. Novel variants are at times associated with completely new histopathological findings, as in, for example, a case of MERRF with a novel MTTK variant that was found to have p62-positive intranuclear inclusions on skin biopsy, something never previously noted in patients with MERRF [102]. A new diagnostic and phenotypic advance in our understanding of MERRF pathophysiology, and thus a potential route for therapy, comes from the examination of the post-transcriptional RNA modifications of the tRNALys implicated in the disease. A quantitative RNA sequencing study of MERRF patients with the m8344A>G mutation found that a N1-methyadenosine (m1A) modification was missing at position 58 in the mitochondrial tRNALys. Restoration of the m1A led to translation elongation and the stability of some nascent chains. This shows that post-transcription modifications of mitochondrial tRNAs regulate the control of gene expression and could potentially be exploited therapeutically [100].

### 10.2. Therapeutic Progress

Therapy for MERRF is currently limited to the symptomatic management of the PME and the muscle weakness, with “mitochondrial cocktails” also being an option for patients. Several innovative therapeutic approaches are being considered for MERRF. In one study, researchers attempted to induce mitochondrial biogenesis in cells with various degrees of heteroplasmy [103]. Two approaches were used: (1) directly by the overexpression of the master regulator PGC-1-α or (2) indirectly using nicotinic acid, which is an NAD+ precursor for the ETC, to improve mitochondrial respiration. This approach led to a slight increase in mitochondrial protein expression and respiration in cell lines with wildtype and intermediate-mutation loads but was ineffective in high-mutation-load cell lines. These results suggest that this approach may not be feasible for patients with a high mutation load. These researchers also attempted to stimulate the removal of damaged mitochondria through the prolonged use of rapamycin, which induces the inhibition of mechanistic target of rapamycin complex 1 (mTORC1) and downstream activation of transcription factor EB (TFEB), which mediates increased mitochondrial biogenesis [103]. This treatment was applied for 4 weeks and did induce a slight increase in mitochondrial respiration in the fibroblasts with a high mutation load. The improvement was even more noticeable in the intermediate mutation load. Overall, this approach suggests that rapamycin could be a therapeutic option for MERRF.

Another interesting approach to the treatment of the mitochondrial disorders MERRF and mitochondrial myopathy, encephalopathy, lactic acidosis, and stroke-like episodes (MELAS) involves so-called “epitranscriptomic” therapies. Researchers have noted significant reductions in the frequency of certain RNA bases (5-taurinomethyluridine, 2-thiouridine derivative) in the anticodons of mutant mt-tRNAs isolated from the cells of patients with these disorders [104]. These hypomodified tRNAs are unable to decode cognate codons effectively, which leads to the defective translation of proteins in the ETC in mitochondria. The overexpression of MTO1, a 5-taurinomethyluridine-modifying enzyme, led to the restoration of the 2-thiouridine derivative of the mutant mt-tRNALys in MERRF patient myoblasts. This suggests that MTO1 may restore the translation of ETC proteins and offers a new path forward to treat these mitochondrial disorders that involve mutations of mt-tRNAs.

Very preliminary work has been performed looking at the use of a bacterial protein called cytotoxic necrotizing factor 1 (CNF1) as a novel strategy for mitochondrial diseases [105]. It acts on the Rho GTPases that regulate actin cytoskeleton. When applied to the fibroblasts of a patient with the typical mutation of MERRF, the cells recovered the wildtype-like mitochondrial morphology and were found to have increased their cellular content of ATP, suggesting a powerful impact on the ETC. The researchers also noted significant F-actin reorganization in these fibroblasts, suggesting that actin organization helps to prevent or limit cellular damage from mitochondrial impairment and the CNF1 may prove beneficial for these patients.

Studies have found that the carboxy-terminal domain (C-term) of human mt-leucyl tRNA synthetase rescues the pathogenicity of the m.3243A>G mutation in mt-tRNALeu or with the mt-tRNAIle [106]. Both mt-tRNAs are amino-acylated by class I mt-aminoacyl-tRNA synthetases (mt-aaRS). A follow-up study showed that the C-term can improve the phenotype caused by the m.8344A>G mutation in mt-tRNALys, which is aminoacylated by a class II aaRS. The same rescue could be achieved by C-term-derived short peptides that have high affinity for the wildtype and mutant human mt-tRNALys. These short peptides are an exciting avenue to rescue cellular defects in patients impacted by MERRF [106].

## 11. Miscellaneous Genetic PMEs

There are quite a few additional PMEs with known genetic causes. However, there are very few publications on these PMEs. We therefore chose to summarize the information on these PMEs in tabular form for ease of access. Please see Table 2 for a list of these rare PMEs and their associated publications.

## 12. Conclusions

The PMEs are a heterogeneous set of genetic disorders with overlapping symptoms of progressive myoclonus and epilepsy. However, each PME has its unique features, as we have hopefully made clear throughout this review. Breakthroughs both in the pathophysiology and in the therapies for certain PMEs, such as LD, ULD, and MERRF are advancing rapidly, including a publication from our group showing evidence in favor of gene therapy in a murine model of ULD [149]. For other PMEs, progress has been more incremental. Lessons learned from certain PMEs are likely to translate into more efficient diagnostic modalities, phenotypic understanding, and therapies for other, perhaps rarer, PMEs. Examples of this include electrophysiologic biomarkers such as giant SSEPs and pronounced PPR, treatments of neuroinflammatory cascades that are common to the PMEs, gene therapies, enzyme replacement therapies, and epitranscriptomic therapies. For such a devastating and challenging set of diseases, the future appears brighter than ever before.

## Figures and Tables

**Table 1:** The most common PMEs with their associated genes and gene products.

PME (Acronym)	Affected Genes	Protein Function
Unverricht-Lundborg disease (ULD)	*CSTB*	Cystatin B/Stefin B, a cathepsin B lysosomal protease inhibitor
Lafora disease (LD)	*EPM2A*, *EPM2B*	Laforin, a glycogen phosphatase; malin, an E3-ubiquitin ligase
Neuronal ceroid lipofuscinosis (NCLs)	Several *CLN* genes	Cln1/Ppt1, lysosomal palmitoyl protein thioesterase; Cln2/Tpp1, lysosomal tripeptidyl peptidase 1; Cln3, a lysosomal membrane protein; others
Sialidosis (ST-1)	*NEU1*	Neu1, lysosomal α-N-acetylneuraminidase
North Sea PME	*GOSR-2*	Golgi SNAP receptor 2
Acid ceramidase deficiency (Farber disease/SMA-PME)	*ASAH1*	Asah1, acid ceramidase
Myoclonic epilepsy with ragged red fibers (MERRF)	*MT-TK*	Mitochondrial lysine transfer tRNA

**Table 2:** A table of rare PMEs with their associated gene names, gene products and function, and associated citations. Gene product descriptions come from the gene data information available through the National Center for Biotechnology Information (NCBI) of the National Institutes of Health [107].

Gene	Protein Function	References
*KCNC1*	A member of the integral membrane proteins that mediates the voltage-dependent potassium ion permeability of excitable membranes	Muona et al. [108] Carpenter et al. [109] Clatot et al. [110,111]
*NEXMIF*	Neurite extension and migration factor	Chorny et al. [111]
*SEMA6B*	Semaphorin 6B, member of the semaphorin family, a group of proteins characterized by the presence of a conserved semaphoring domain, which typically play a role in axon guidance	Herzog et al. [112] Duan et al. [113] Hamanaka et al. [114] Chen et al. [115]
*GALC*	Galactosylceramidase, a lysosomal protein that hydrolyzes the galactose ester bonds of galactosylceramide; associated with Krabbe disease	Shang et al. [116]
*SCARB2*	Scavenger receptor class B member 2, a type III glycoprotein found mostly in limiting membranes of lysosomes and endosomes; it may participate in membrane transportation	Quraishi et al. [117] Atasu et al. [118] Yari et al. [119]
*KCTD7*	A member of the potassium channel tetramerization domain-containing protein family	Dudipala et al. [120] Narayanan et al. [121] Farhan et al. [122]
*NUS1*	NUS1 dehydrodolichyl diphosphate synthase subunit, a type I single transmembrane domain receptor, which is a subunit of cis-prenyltransferase and serves as a specific receptor for the neural and cardiovascular regulator Nogo-B	Araki et al. [123] Riboldi et al. [124]
*PRICKLE1*	Prickle planar cell polarity protein 1, nuclear receptor that may be a negative regulator of the Wnt/β-catenin signaling pathway	Ban et al. [125] Algahtani et al. [126]
*ATP6V0A1*	ATPase H+ transporting V0 subunit A1, encodes a component of vacuolar ATPase (V-ATPase), a multisubunit enzyme that mediates acidification of eukaryotic intracellular organelles	Bott et al. [127]
*LMNB2*	B-type nuclear lamin	Farajzadeh et al. [128]
*DHDDS*	Dehydrodolichyl diphosphate synthase subunit, catalyzes cis-prenyl chain elongation to produce the polyprenyl backbone of dolichol	Kim et al. [129]
*CYP27A1*	Cytochrome P450 family 27 subfamily A member 1	Desai et al. [130]
*MT-ND6*	Mitochondrially encoded NADH dehydrogenase 6, enables NADH dehydrogenase (ubiquinone) activity	Khoo et al. [131]
*ARV1*	Transmembrane protein that contains a conserved zinc ribbon motif at the N-terminus	Darra et al. [132]
*GBA*	Glucosylceramidase, a lysosomal membrane protein that cleaves the β-glucosidic linkage of glycosylceramide	Poffenberger et al. [133] Tonin et al. [134]
*BSCL2*/Seipin	Multi-pass transmembrane protein called seipin, which localizes to the endoplasmic reticulum and may be important for lipid droplet morphology	Pedicelli et al. [135] Opri et al. [136]
*SERPINI1*	Serpin family 1 member 1, member of the serpin superfamily of serine proteinase inhibitors	Kara et al. [137]
*KCNA2*	Potassium voltage-gated channel subfamily A member 2	Canafoglia et al. [138]
*SZT2*	SZT2 subunit of KICSTOR complex, expressed in the brain; localizes to the peroxisome and is implicated in resistance to oxidative stress	Domingues et al. [139]
*CERS1*	Ceramide synthase 1, which catalyzes the synthesis of ceramide, the hydrophobic moiety of sphingolipids	Godeiro Junior et al. [140]
*GPR37L1*	G-protein-coupled receptor 37 like 1, a protein that enables G-protein-coupled peptide receptor activity, peptide binding activity, and prosaposin receptor activity	Giddens et al. [141]
*CACNA1A*	Calcium voltage-gated channel subunit alpha1 A	Sun et al. [142] Lv et al. [143]
*FARS2*	Phenylalanyl-tRNA synthetase 2 (mitochondrial)	Walker et al. [144]
*GSTA1, GSTM1, GSTP1, GSTT1*	Glutathione S-transferases, a family of enzymes that function to add glutathione to target electrophilic compounds	Ercegovac et al. [145]
*SACS*	Encodes the protein sacsin, responsible for Spastic Ataxia of Charlevoix-Saguenay	Nascimento et al. [146]
*IRF2BPL*	Interferon regulatory factor 2 binding protein like, which is a transcriptional regulator in the central nervous system	Gardella et al. [147] Costa et al. [148]
